# Epidemiological Patterns of Common Cancers in Costa Rica: An Overview up to 2020

**DOI:** 10.4236/jss.2023.116033

**Published:** 2023-06-29

**Authors:** Alejandro Calderon, Percy Guzman, John D. Murphy

**Affiliations:** 1 Caja Costarricense del Seguro Social, San Jose, Costa Rica; 2 Cancer Prevention Fellowship Program (CPFP), Division of Cancer Prevention (DCP), National Cancer Institute (NCI), Maryland, United States; 3 Health Assessment Research Branch (HARB), Health Delivery Research Program (HDRP), Division of Cancer Control and Population Sciences (DCCPS), Maryland, United States; 4 Metabolic Epidemiology Branch (MEB), Division of Cancer Epidemiology and Genetics (DCEG), Maryland, United States

**Keywords:** Epidemiological Patterns, Cancers, Costa Rica

## Abstract

**Introduction:**

The increasing cancer incidence and mortality rates in Costa Rica have become a public health concern, with prostate, breast, and colorectal cancers being the most prevalent. This study aimed to analyze the trends in cancer incidence and mortality rates by tumor type, sex, age group, and geographic region using data from the Costa Rican National Cancer Registry.

**Methods:**

In this retrospective study, we analyzed cancer incidence and mortality anonymized data from the Costa Rican National Cancer Registry between 2010 and 2020. The study included data on cancer cases diagnosed and deaths attributable to cancer within the population.

**Results:**

Our findings revealed variations in cancer incidence and mortality rates based on geographic region, age group, and tumor type. Cancer was most predominant in the province of San Jose, followed by Alajuela, Heredia, Cartago, Guanacaste, and Puntarenas. Breast cancer, colorectal cancer, and prostate cancer were the leading causes of cancer in both sexes. Mortality rates due to gastric cancer and prostate cancer were highest among men, while breast cancer was the leading cause of cancer mortality among women.

**Conclusion:**

The results of this study underscore the need for targeted prevention and screening programs, improved access to treatment for patients living in rural areas, and a comprehensive cancer control program in Costa Rica. By implementing evidence-based interventions, such as tobacco control programs, cancer screening initiatives, and equitable access to cancer treatment, the cancer burden in Costa Rica can be mitigated, ultimately improving the overall health of the population.

## 1. Introduction

Cancer is a significant public health concern worldwide, responsible for a large proportion of deaths each year. According to recent estimates, there were 19.3 million new cancer cases and 9.96 million cancer deaths globally in 2020, with projections showing that these numbers will rise to 30.2 million new cases and 16.3 million deaths by 2040 (Bray et al., 2015). The burden of cancer is especially high in low- and middle-income countries (LMICs), with over 70% of cancer deaths occurring in these regions (WHO, 2021). Early detection of cancer is crucial to preventing and treating the disease, and Costa Rica has implemented several cancer screenings programs, including breast and cervical cancer screening, to improve early detection rates (Lindsay et al., 2020). However, access to screening services remains a challenge, particularly for underserved populations (Lindsay et al., 2020).

Cancer is expected to become the leading cause of death globally by 2040, with higher incidence and mortality rates in men than in women (Bray et al., 2018). In Latin America, cancer incidence and mortality rates are projected to rise, with more than 60% of incident cancers occurring in this region (IARC, 2020). The most frequently diagnosed cancers in men were prostate, colorectal, lung, stomach, and bladder cancers, while breast, colorectal, cervical, thyroid, and lung cancers were the most common in women (IARC, 2020). The highest mortality rates in men were from prostate, lung, colorectal, stomach, and liver cancers, and in women were from breast, lung, colorectal, cervix, and stomach cancers (IARC, n.a.).

Despite having a national cancer registry, Costa Rica has made little effort to disseminate its incidence data widely among a scientific audience. Therefore, the aim of this study is to provide an overview of the epidemiology of cancer in Costa Rica up to 2020, investigating changing trends in the incidence and mortality rates of specific types of cancer over time and identifying demographic or regional differences in cancer incidence. In addition, this study will expand on the discussion section, which was previously summarized in the results section, by exploring the impact and relevance of the results demonstrated, the evolving systemic treatment scenario for common tumors, and the limitations of the current report. Recent papers will also be added for consistency (Williams et al., 2021; Waks et al., 2021; Zhang et al., 2021; Kwon & Kim, 2021). This study has the potential to inform public health policies and interventions for cancer prevention and treatment in Costa Rica and other LMICs.

## 2. Methodology

### 2.1. Research Question and Hypothesis

The aim of this study is to investigate changing trends in the incidence and mortality rates of specific types of cancer over time, and to identify demographic or regional differences in cancer incidence in Costa Rica.

### 2.2. Data Sources

**Figure 1.**
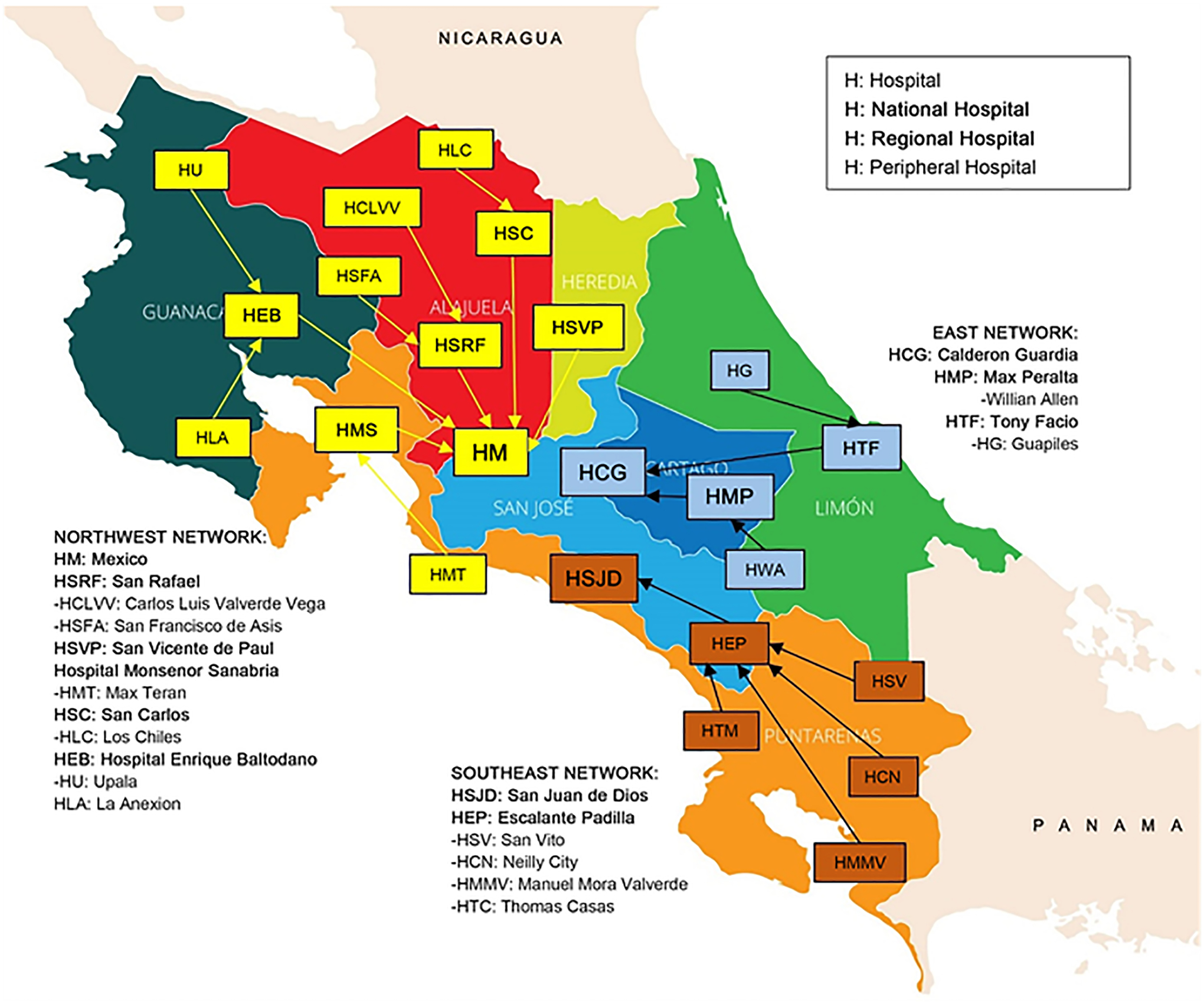
CR map and distribution of hospitals nationwide.

To address the research question and hypothesis, we conducted a comprehensive review of several sources, including the National Tumor Registry (NTR), Ministry of Health, National Institute of Statistics and Censuses (NISC), see Figure 1, World Health Organization (WHO), and International Agency for Research on Cancer (IARC) websites (Ministerio de Salud, 2021; WHO, 2021; IARC, 2020).

### 2.3. Data Limitations

However, the available data had major issues, as the Costa Rican registries had limited information available only up to 2015 on mortality and 2014 on incidence. To supplement this information, we used the data up to 2020 available on the IARC website (IARC, 2020). It should be noted that the numbers and rates reported by the registry are based on topography of the ICD-O-3 classification without converting to ICD-10, which is the standard to present cancer registry data by site (WHO, 2010). This can affect the results, especially for lymphoma and leukemia. Therefore, we took care to consider the reliability and comparability of the data obtained.

### 2.4. Data Analysis

To investigate the research question and hypothesis, we analyzed the NTR and IARC data available up to 2020, covering a total of 141,579 cases of cancer, using appropriate statistical tests and models. We used chi-square tests to compare differences in cancer incidence and mortality rates between sexes and age groups. We also calculated crude incidence and mortality rates by cancer site and age group, and standardized incidence and mortality rates per 100,000 person-years using the World Standard Population (WHO, 2021). To identify tem-poral trends in cancer incidence and mortality rates (Kwon & Kim, 2021). Finally, we examined changes in incidence and mortality rates over time for specific types of cancer and identified demographic or regional differences in cancer incidence.

### 2.5. Analytical Tools

To provide insights into the geographic distribution of cancer burden, we described the incidence and mortality in the five cantons with the highest rates in each province. For this analysis, we used Geographic Information Systems (GIS) software.

### 2.6. Data Presentation

All incidence and mortality rates were presented per 100,000 individuals. To prepare the graphs and figures, we used appropriate statistical software such as SPSS version 17.0.1 and STATA.

### 2.7. Summary of Methods

The methods section provides a clear overview of the research question and hypothesis, data sources and limitations, analytical tools and software, and data presentation. The section explicitly states the statistical tests and models used for data analysis, and the use of GIS software for analyzing the geographic distribution of cancer burden. The section also emphasizes the importance of considering the reliability and comparability of the data obtained.

## 3. Results

### 3.1. Cancer Incidence in Costa Rica

The incidence of cancer in Costa Rica has been increasing since the 1990s. In 1990, the incidence rate was 135.1 per 100,000, while in 2020, it was 188.7 per 100,000, representing a 72% increase in 30 years. In 2020, there were 13,139 new cancer cases reported in the country.

In 2020, Costa Rica had the highest incidence of cancer in Central America with an incidence rate of 188.7 per 100,000 in both sexes. Nicaragua (135.7), Honduras (133.7), El Salvador (129.7), Guatemala (123.1), and Belize (120.9) followed in second to sixth place, respectively.

**Figure 2.**
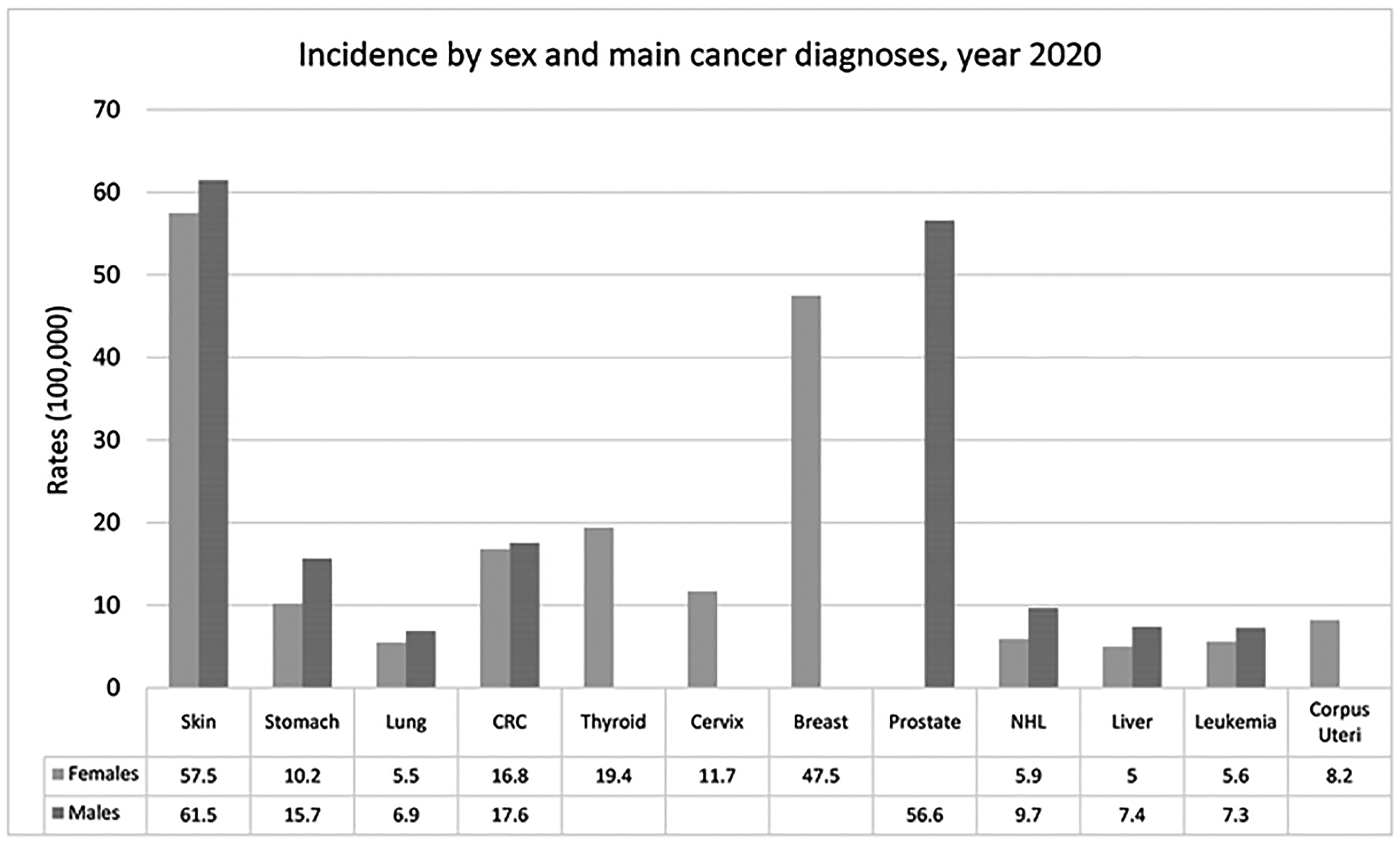
Incidence of main cancers by sex in the year 2020.

The ranking of incidence by tumor type in 2020 for both sexes was as follows (see Figure 2): prostate cancer had the highest incidence rate (56.6), followed by breast cancer (47.5), colorectal cancer (17.2), non-melanoma skin cancer (13.5), stomach cancer (12.8), and cervical cancer (11.7). The group of skin cancer, melanoma, and non-melanoma skin cancer (NMSC) were presented together in Figure 2 at an incidence rate of 61.5 in men and 57.5 in women.

**Figure 3.**
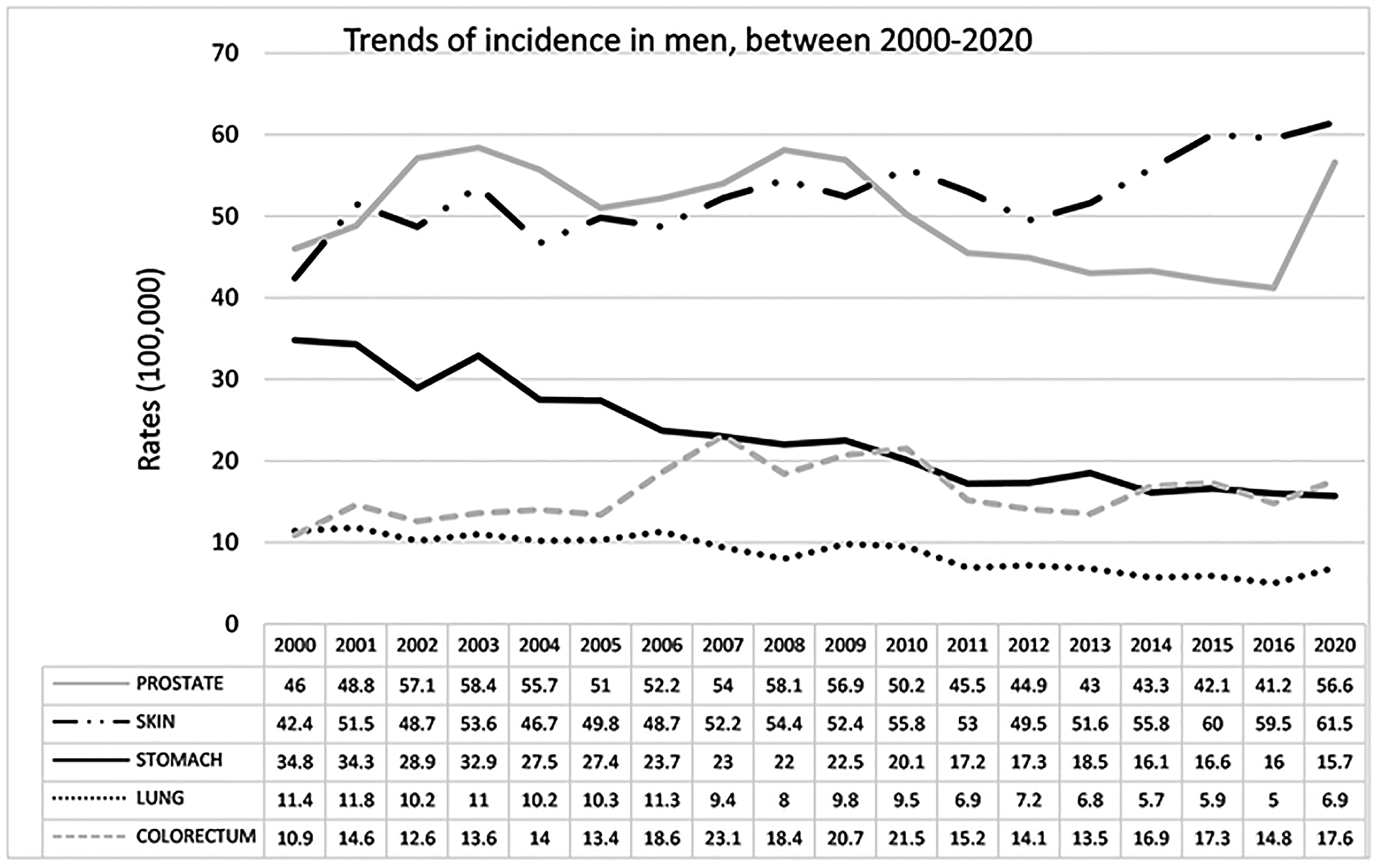
Trends of cancer incidence in men between 2000 and 2020.

In men, the incidence trends in the year 2000 placed prostate cancer in first place (46.0), followed by skin cancer (42.4), stomach cancer (34.8), lung cancer (11.4), colorectal cancer (10.9), and lymph nodes (e.g., non-Hodgkin lymphoma, Hodgkin lymphoma) (5.6). Skin and colorectal cancers showed an increasing trend during the period 2000–2020, whereas there was a decreasing trend in prostate, stomach, and lung cancers. Skin cancer increased by 40% in this period, displacing prostate cancer (56.6 in 2020) in second place. Colorectal cancer (17.6 in 2020) showed a constant increase throughout the study period (35% increase in the period), going from fifth to fourth place. Stomach cancer (15.7 in 2020) presented a substantial decrease (55%), remaining in third place. Lung cancer decreased by 39% from fourth place in 2000 to sixth place in 2020 (6.9). Non-Hodgkin lymphoma (NHL) was ranked fifth in 2020, with a rate of 6.9 (see Figure 3).

**Figure 4.**
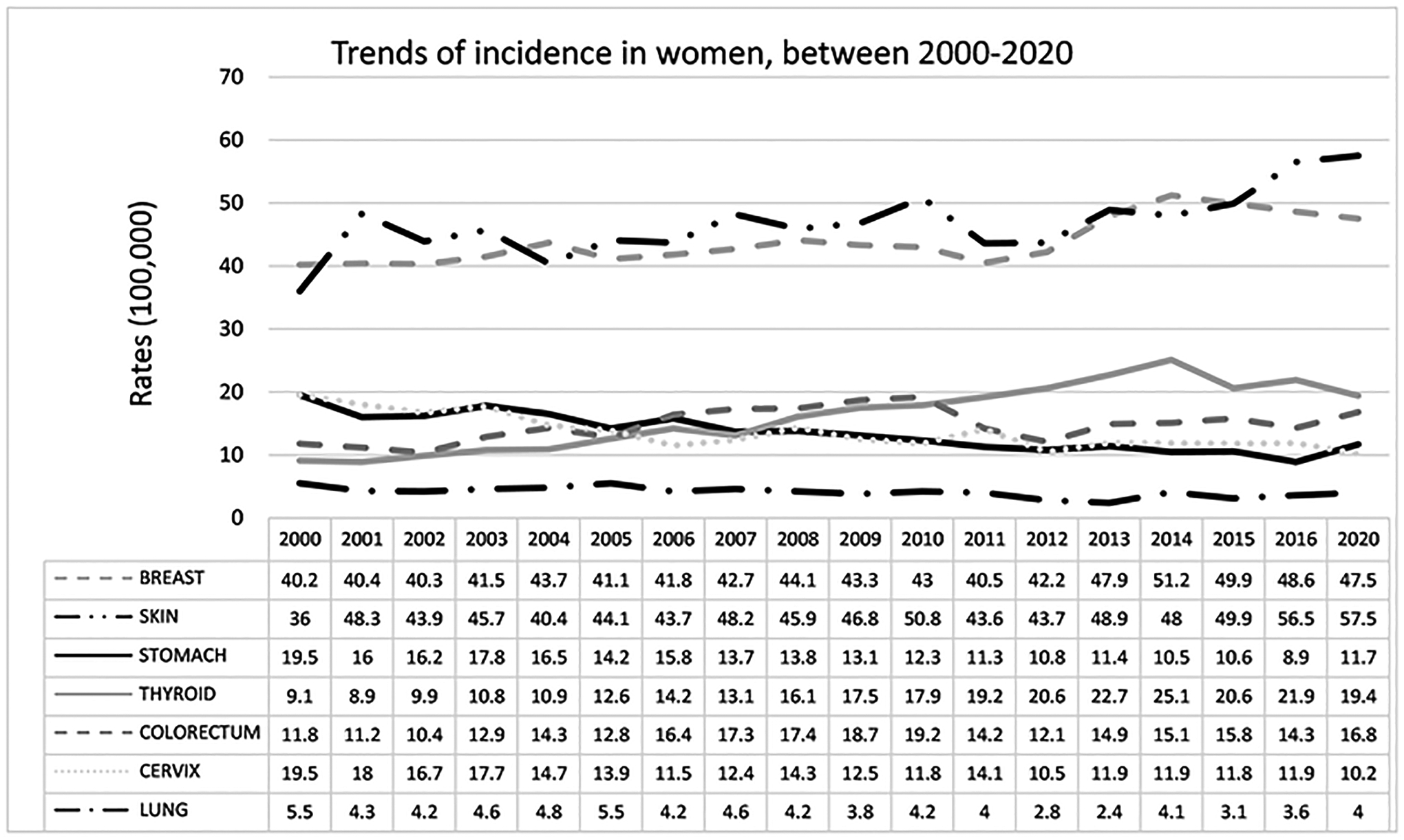
Trends of cancer incidence in women between 2000 and 2020.

In women, the highest rates in the year 2000 were observed in breast (40.2), skin (36.0), stomach (19.5), cervical (19.5), colorectal (11.8), thyroid (9.1), and lung (5.5) cancers. The incidence of cervical, stomach, and lung cancers showed a decreasing trend, whereas that of melanoma, breast cancer, colorectal cancer, and thyroid cancer increased in the period 2000–2020. Cervical cancer decreased by 40% in the period (19.5 in 2000 to 11.7 in 2020) and stomach cancer also decreased by 47%, showing a decreasing trend from 19.5 in 2000 to 10.2 in 2020, going from fourth place in 2000 to fifth place in 2020. Lung cancer incidence has shown a slight decrease from 5.5 in 2000 to 4.0 in 2020. Skin cancer went from 36 in 2000 to 57.5, an increase of 58%. The incidence of breast cancer increased from 40.2 in 2000 to 47.5 new cases in 2020, an increase of 21%. CRC declined from 11.8 (5th place) in 2000 to 16.8 (3rd place) in 2020. Thyroid cancer increased by about 135%, from 9.1 in 2000 to 19.4 in 2020, from 6th place in 2000 to third place in 2020, see Figure 4.

#### 3.1.1. Incidence by Age Group

**Figure 5.**
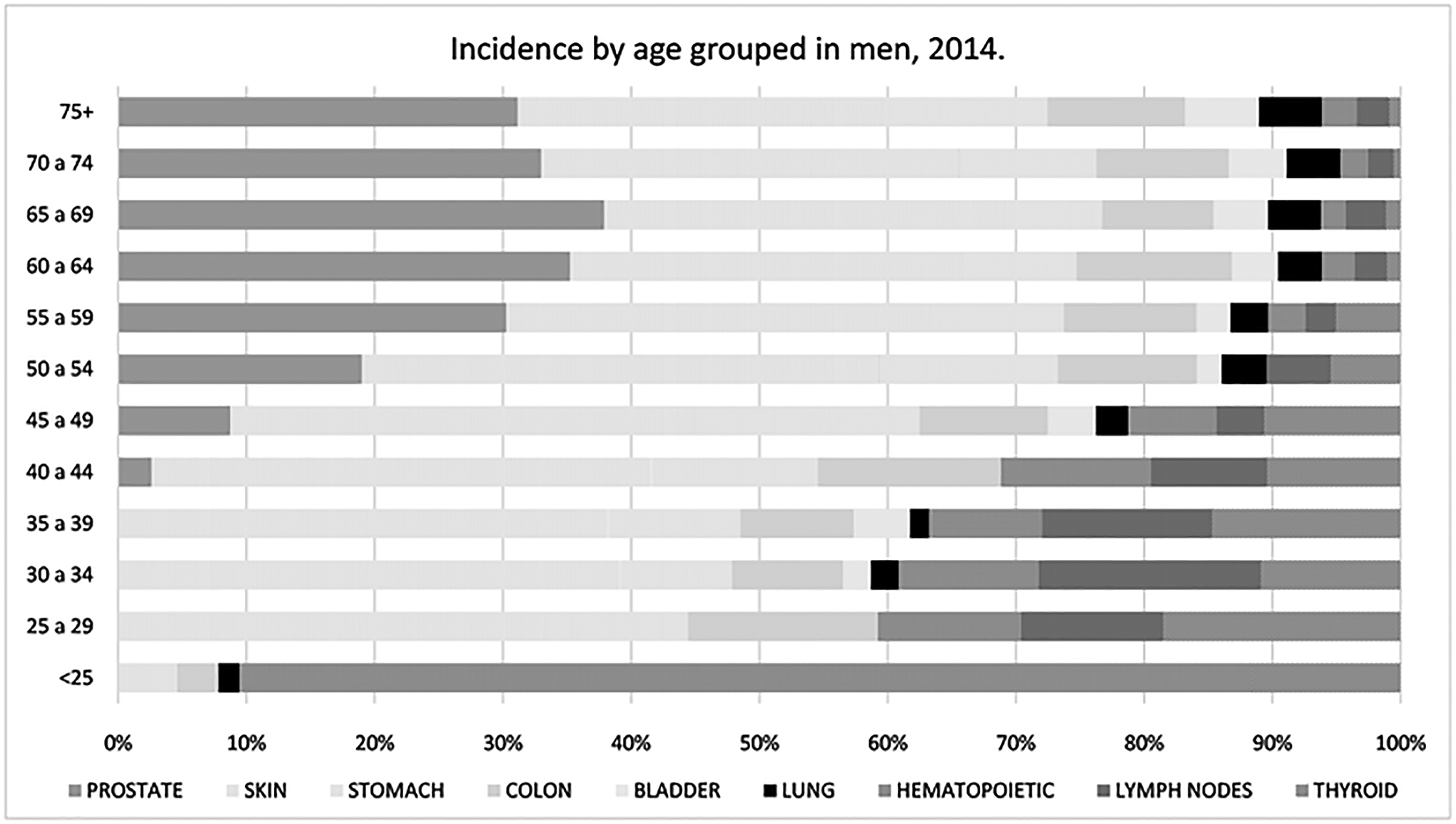
Age specific incidence rates of main cancers in men in 2014.

The incidence rates in 2014 in men aged < 25 years were as follows: hematopoietic (22.82), lymphatic (11.67), thyroid (3.36), skin (1.32) and colon cancers (0.94). Among patients aged between 25 and 60 years, skin cancer was the most common. The incidence of gastric cancer has been shown to increase among patients > 40 years. The incidence of prostate ranked first in patients aged > 60 years, see Figure 5.

**Figure 6.**
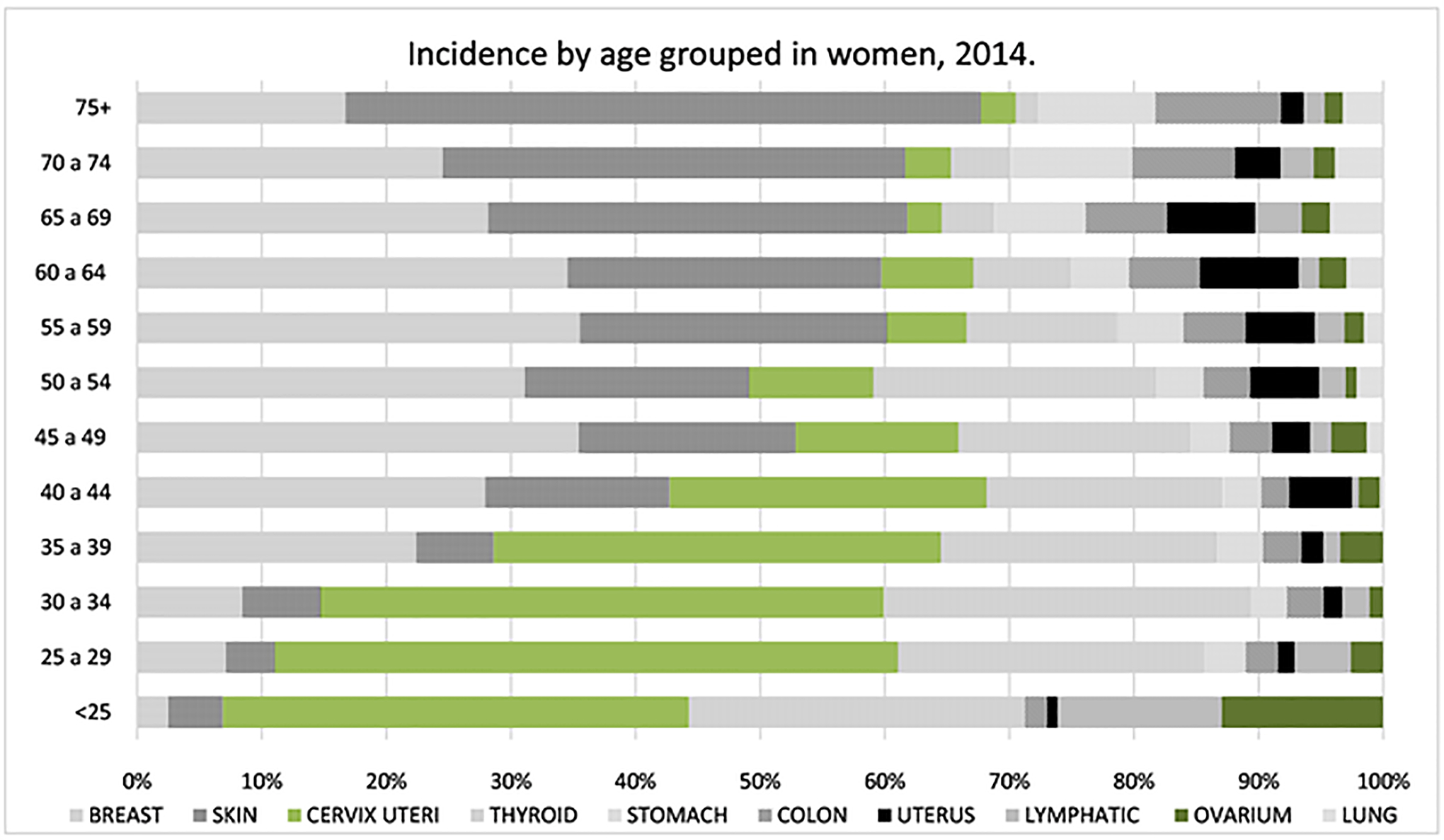
Age specific incidence rates of main cancers in women in 2014.

**Figure 7.**
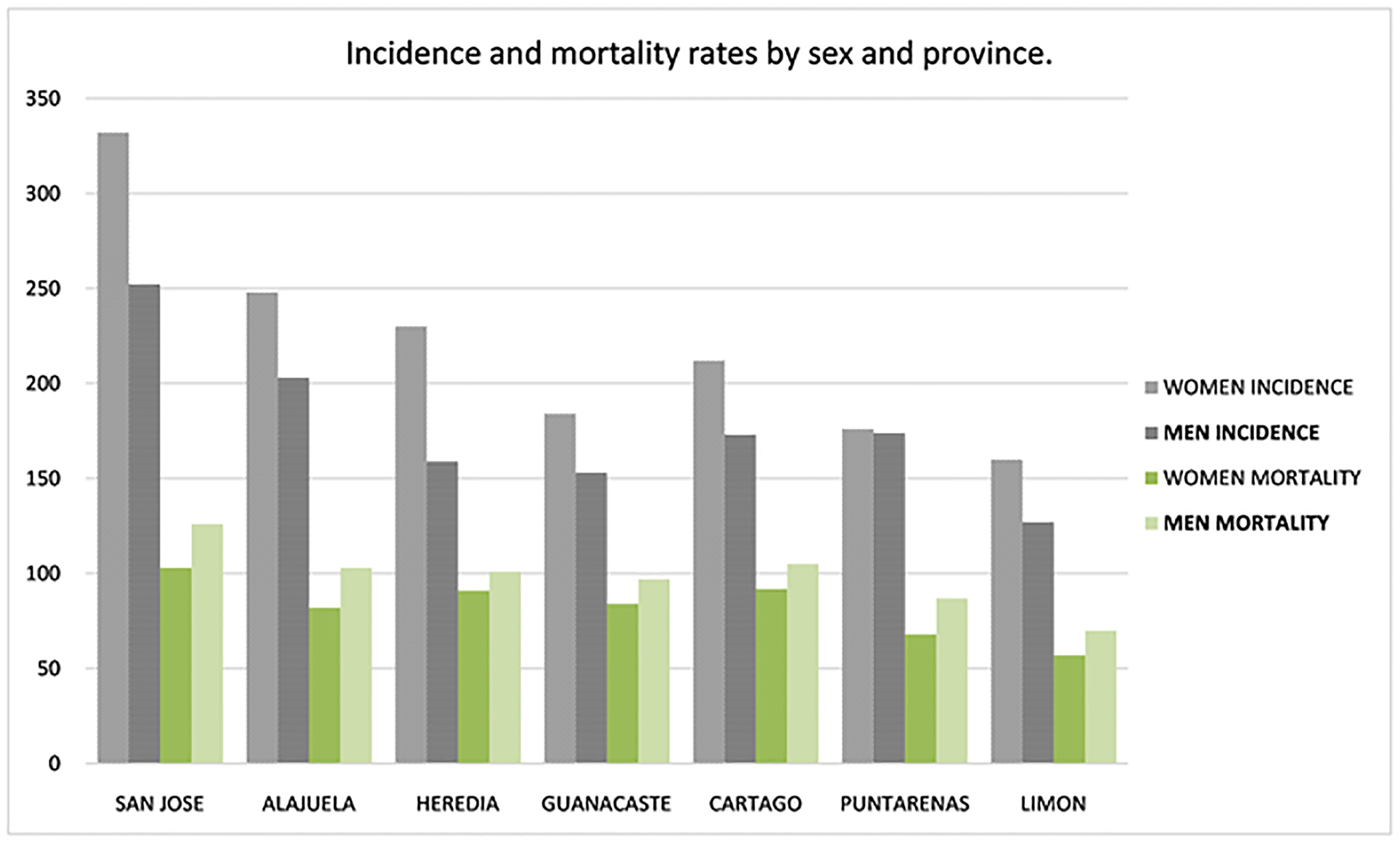
Cancer incidence and mortality rates by sex and province.

Among women aged < 25 years in 2014, the highest cancer incidence rates were observed in the skin (2.54), breast (1.42), and CRC (1.03). Cervical cancer has the highest incidence (180.7) in patients aged 15 – 40 years of age. Breast cancer had the highest incidence (713.57) among the group of patients aged 40 – 65 years. For patients over 70 years of age, skin cancer (948.14) was the most common, followed by breast (426.04), CRC, stomach, and lung cancer, see Figure 6 Incidence by sex and province (Figure 7).

The incidence rate in men was 194.1 (6521 cases; 49.6% of all cancer cases) and in women it was 186.0 (6618 cases or 50.4% of cases).

The distribution of incidence by sex and canton was only available until 2014 in the NTR. Among men, the province of San José had the highest cancer incidence rate (252). The cantons with the highest rates were downtown San José (374), Puriscal (324), Moravia (305), Perez Zeledón (288) and Coronado (282). The province of Alajuela ranked second at a rate of 203, including San Mateo (345), Orotina (317), Atenas (301), downtown Alajuela (246) and San Ramón (226). The province of Puntarenas ranked third with a cancer incidence rate of 174, among which the canton rates were Montes de Oro (266), Aguirre (243), Coto Brus (217), Esparza (207) and downtown Puntarenas (172). Cartago, fourth place, presented 173 cases per 100,000 men; the rates among its cantons were Turrialba (261), downtown Cartago (212), Jiménez (158), Paraíso (146) and Oreamuno (135). Heredia in fifth place, had a rate of 159, canton rates included: Belén (255), San Isidro (228), Santo Domingo (207), San Rafael (186) and Santa Barbara (167). Guanacaste, in sixth place, reported rates of 153, and its cantons were Tilarán (227), Liberia (201), Abangares (194), Bagaces (177) and Carrillo (157). Finally, Limón showed rates of 127, including downtown Limón (155), Guácimo (153), Pococí (143), Siquirres (125) and Talamanca (49).

In women, cancer was predominant in the province of San José with rates of 332. Cantons with the highest incidence rates were downtown San José (454), Puriscal (402), Pérez Zeledón (393), Turrubares (366) and Montes de Oca (339). Alajuela ranked second at a rate of 248, including Atenas (370), San Mateo (309), Orotina (306), downtown Alajuela (275) and Grecia (270). Heredia, third, had a provincial rate of 230, including Santo Domingo (291), downtown Heredia (286), Belén (279), Flores (242), and San Isidro (241). Fourth place Cartago observed 212 cases and included Turrialba (279), Jiménez (243), downtown Cartago (236), Alvarado (221) and Paraíso (191). Guanacaste, in fifth place, presented rates of 184, including Liberia (254), Tilarán (241), Nandayure (236), Hojancha (214) and Cañas (206). Puntarenas was ranked sixth at a rate of 176, its cantons were Montes de Oro (284), downtown Puntarenas (225), Coto Brus (193), Esparza (165) and Aguirre (141). Finally, Limón reported rates of 169, including Pococí (205), downtown Limón (158), Matina (150.2), Guácimo (150) and Siquirres (132).

Among the provinces, San José had the highest incidence of cancer in both sexes combined, followed by Alajuela, Heredia, Cartago, Guanacaste, Puntarenas, and Limón. The incidence rate in the country’s capital (San José) was 252 per 100,000 inhabitants (3903 new cases), followed by Alajuela (203 per 100,000, 1559 new cases), and Heredia (159 per 100,000, 726 new cases). The incidence rate in Cartago was 173 per 100,000 inhabitants (738 new cases), in Guanacaste it was 153 per 100,000 inhabitants (504 new cases), in Puntarenas it was 174 per 100,000 inhabitants (860 new cases), and finally in Limón it was 127 per 100,000 inhabitants (322 new cases), see Figure 7.

#### 3.1.2. Cancer Mortality in Costa Rica

In Central America, the mortality rates in both sexes showed 81.3 in Honduras, in CR 80.1 in Nicaragua 78.0, in Guatemala 70.7, in El Salvador 66.8, and Belize 66.4.

According to data from the NTR of the Ministry of Health and the NISC, cancer ranks second in mortality, surpassed only by cardiovascular diseases (cardiac ischemia, stroke, etc.). Approximately 50% of deaths from chronic NCDs among people between the ages of 30 and 69 are due to cancer.

According to the WHO and IARC, the cancer mortality rate for CR in 2020 was 118.3 (6028 cases), with sex-specific rates of 125.3 (3189 cases) in men and 111.4 (2839 cases) in women.

#### 3.1.3. Ranking of Mortality by Type of Tumor

**Figure 8.**
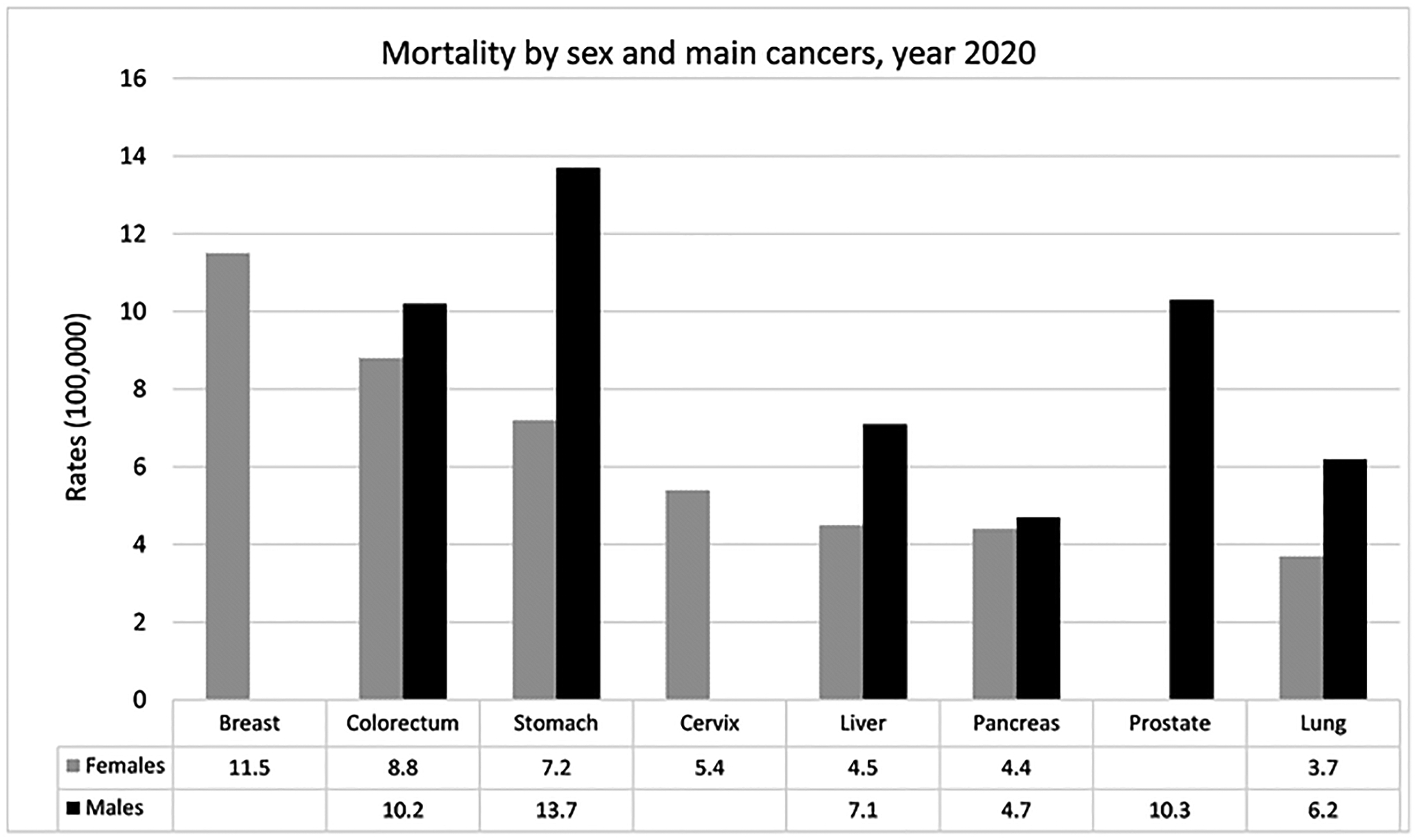
Mortality rates by sex and primary cancers in the year 2020.

**Figure 9.**
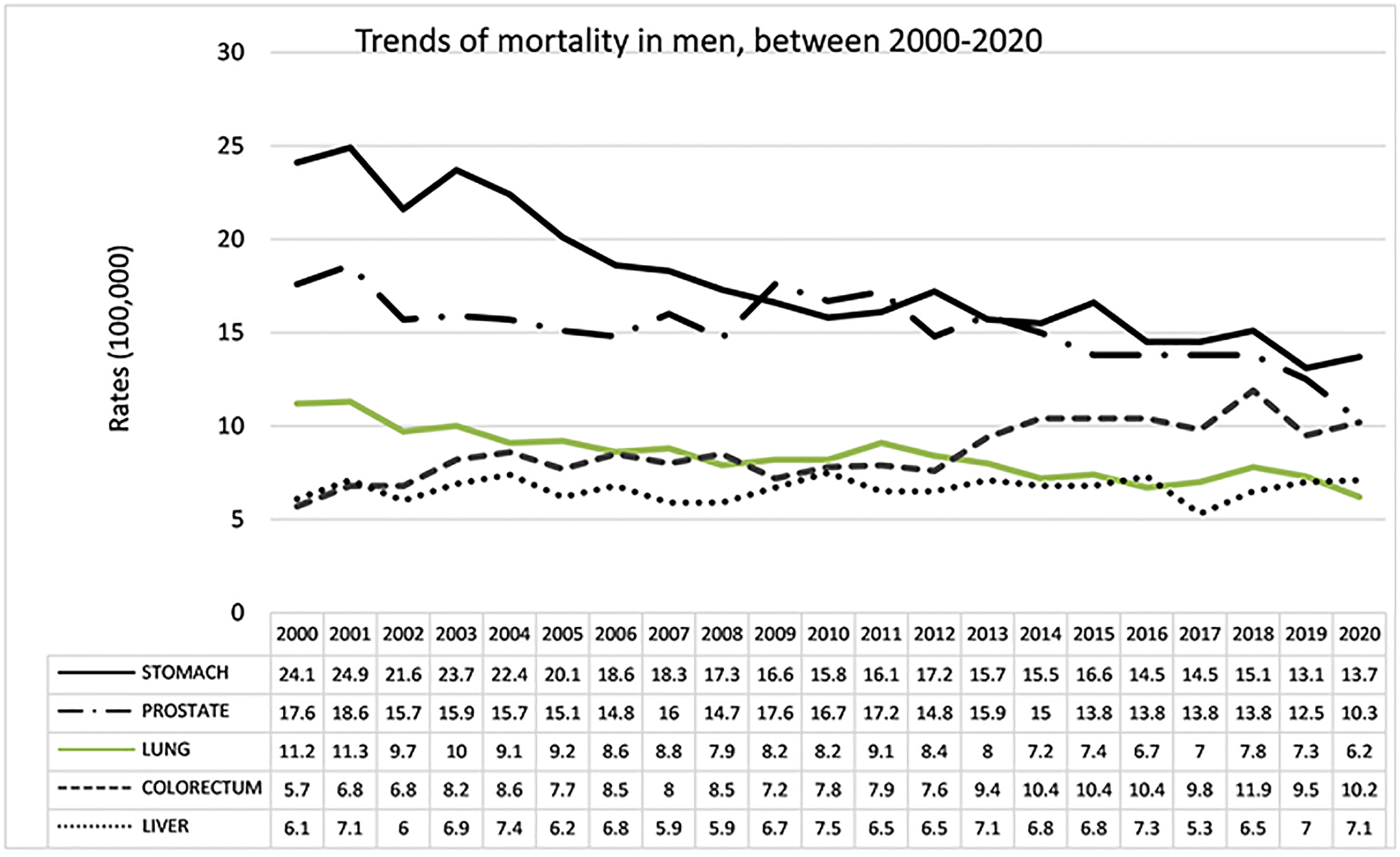
Trends of cancer mortality in men, between 2000 and 2020.

The distribution of deaths (percentage) from cancer in men in 2020 was follows: gastric cancer (13.7%) first, prostate cancer (10.3%), CRC (10.2%), liver cancer (7.1%) and lung cancer (6.2%), see Figure 8. CRC and the liver showed an increase and stomach, prostate and lung showed a decrease in the period 2000–2020. CRC showed an increase of 82%, going from fifth to third place, with rates of 5.7 in 2000 and 10.2 in 2020. Liver cancer presented an increase of 15%, with rates of 6.1 in the year 2000 and 7.1 in 2020, thus moving from sixth to fourth place. The mortality of stomach cancer went from 24.1 in 2000 to 13.7 in 2020, decreasing by 42%. The rate of prostate cancer was 17.6 in 2000 and 10.21 in 2020, a 42% reduction over thirty years. Lung cancer showed a decrease from 11.2 in 2000 to 6.2 in 2020, falling by 44%, moving from third to fourth place in deaths, see Figure 9.

**Figure 10.**
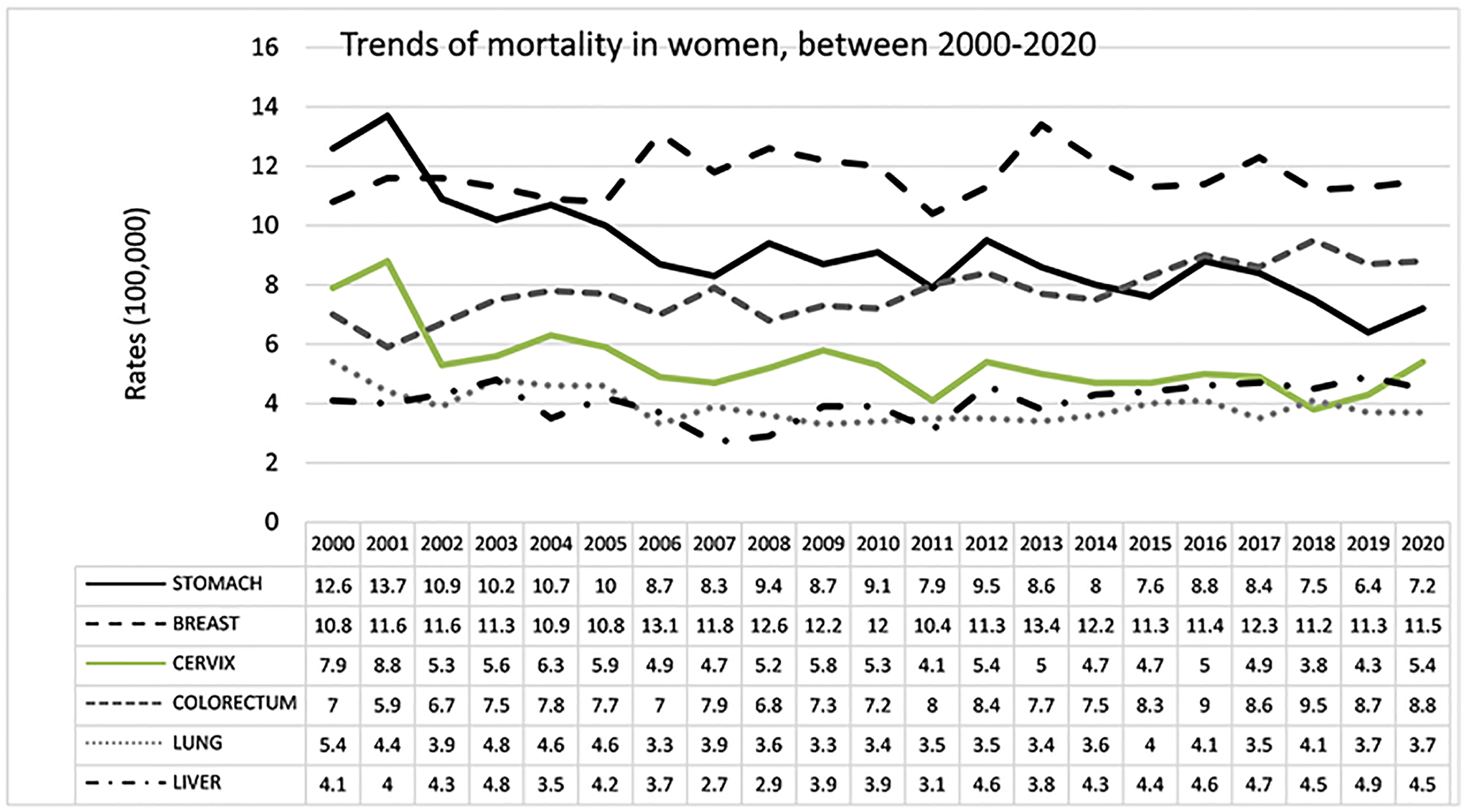
Trends of cancer mortality in women, between 2000 and 2020.

Among women, the highest percentage of deaths from cancer in 2020 was from breast cancer (11.5%), followed by CRC (8.8%), gastric cancer (7.2%), liver cancer (4.5%), and lung cancer (3.7%) in fifth place, see Figure 8. Breast, CRC, and liver cancer showed increases, whereas stomach, cervical and lungs cancer showed decreases in the period 2000–2020. Breast cancer has shown an increase of 6.5%, going from second place to becoming the leading cause of death, with rates of 10.8 in 2000 and 11.5 in 2020. CRC showed a 25% increase, from 7.0 in 2000 to 8.8 in 2020, moving from fourth to second place. The liver showed a 10% increase, from 4.1 per 100,000 in 2000 to 4.5 in 2020. The stomach decreased by 43% from 12.6 in 2000 to 7.2 in 2020, moving from first to third place. The cervix showed rates of 7.9 in 2000 and 5.4 in 2020, a decrease of around 31% from third to fourth place. Lung cancer incidence decrease from 5.4 in 2000 to 3.7 in 2020, representing a 30% decrease from the fifth place in 2000 to the sixth place in 2020, see Figure 10.

#### 3.1.4. Cancer Mortality by Age Group

**Figure 11.**
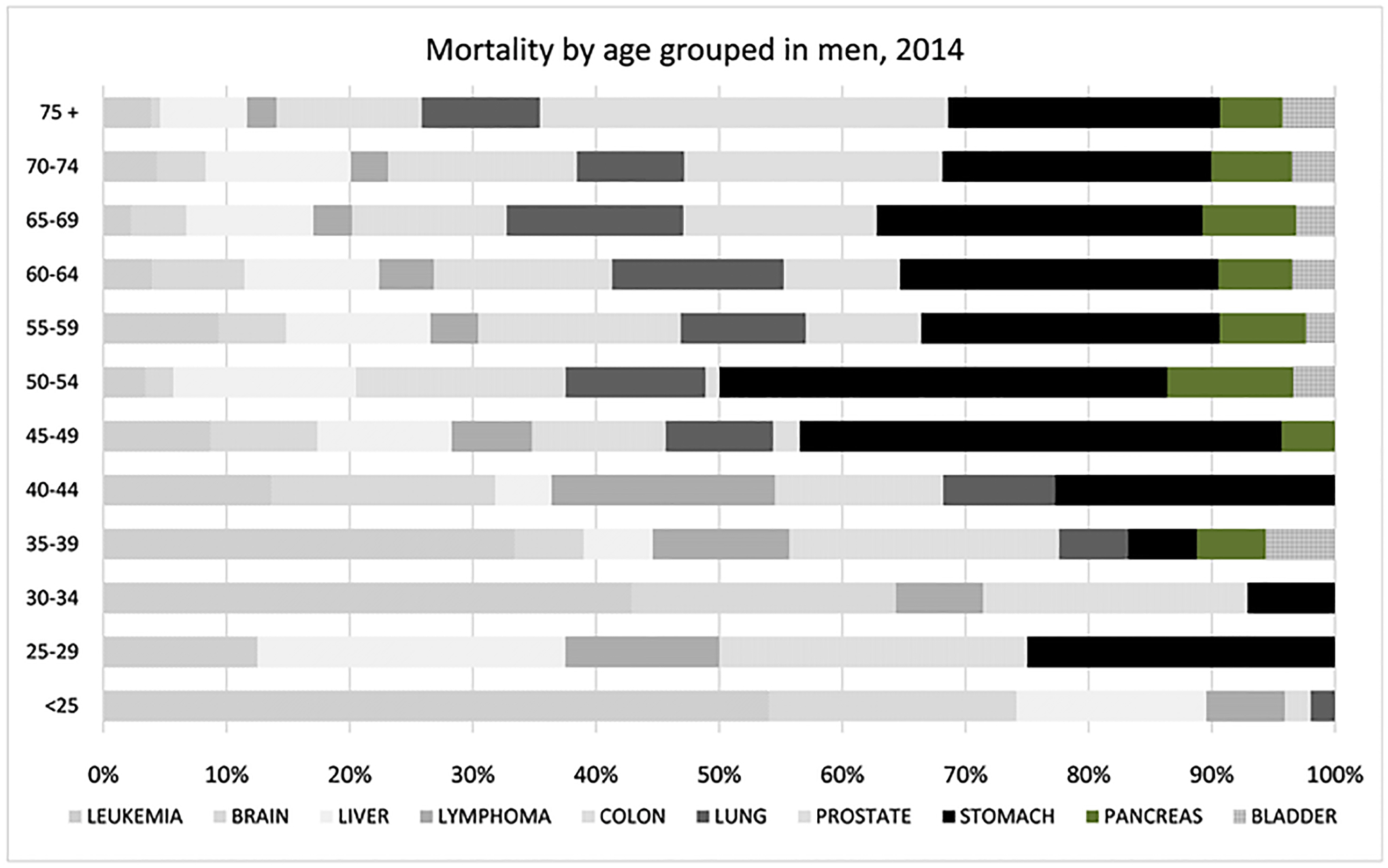
Cancer mortality in men by age group in 2014.

Men aged less than 25 years who died from cancer in 2015 predominantly died from liquid tumors, such as leukemia (11.99), liver (3.42), brain (4.46), and CRC (0.47). Stomach cancer first emerged in patients aged between 40 and 75 years. In patients aged > 75 years, the highest mortality was observed in the prostate (425.53), followed by the stomach (283.68), CRC (151.2), and lung (123.14). The liver had the third highest mortality rate among men aged 45 – 59 years, see Figure 11.

**Figure 12.**
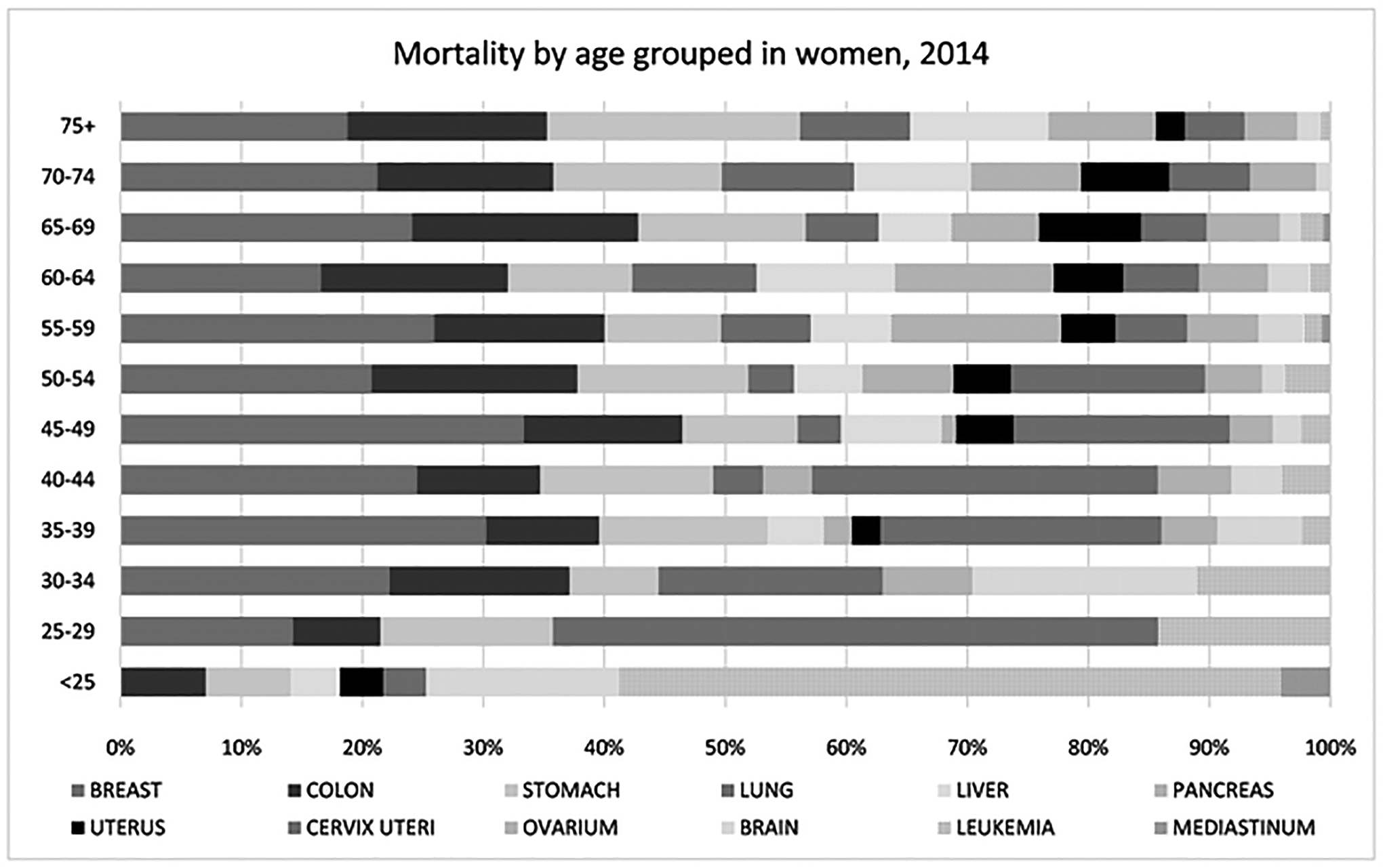
Cancer mortality in women by age group in 2014.

Regarding mortality rates in 2015 for women, those aged < 25 years mainly died from leukemia (7.37), brain cancer (2.16), stomach cancer (0.95), and CRC (0.95). In women aged between 25 and 50 years, we observed premature deaths in the cervix uteri (29.6) and in breast cancer (37.05). In patients aged > 55 years, death from cervical cancer dropped to the sixth place. In this group (>55 years), breast cancer was the most common cause of mortality (321.07), followed by CRC (252.28), stomach (252.10), liver (153.88), and lung (141.57), see Figure 12.

#### 3.1.5. Mortality by Sex and Province

Data on cancer deaths by sex and cantons were available only up to 2015. Among men, cancer mortality was predominant in the province of San Jose, with a rate of 126. In San Jose, the cantons with the highest mortality rates were Leon Cortes (180), Escazu (149), Moravia (148), Turrubares (147), and Puriscal (146). Cartago, the province in second place, had a rate of 105, including cantons Jimenez (145), Oreamuno (133), Turrialba (129), Guarco (111), and downtown Cartago (107). Alajuela, in third place with a rate of 103, included the cantons San Mateo (199), Atenas (155), Naranjo (133), San Rafael (118), and downtown Alajuela (116). Heredia, fourth place, had a rate of 101 and included San Isidro (135), Barva (127), San Rafael (122), Santo Domingo (119) and Flores (118). Guanacaste, in fifth place, showed a rate of 97 and comprised Nandayure (198), Nicoya (142), Tilaran (132), Bagaces (122), and Santa Cruz (104). The province of Puntarenas was in sixth place at a rate of 174. It includes Montes de Oro (132), Aguirre (110), downtown Puntarenas (100), Golfito (98), and Buenos Aires (90). Finally, Limón had a rate of 70, comprising Siquirres (67), Pococi (63), downtown Limón (59), Guacimo (58) and Talamanca (44).

Among women, cancer mortality was predominant in the province of San José, at a rate of 103. The cantons in San Jose with the highest cancer mortality among women were the Leon Cortes (190), Mora (139), Curridabat (132), Acosta (128), and Puriscal (118). Cartago, in second place, observed a rate of 92 and included Alvarado (124), Guarco (115), Oreamuno (104), downtown Cartago (101) and Turrialba (88). In third place, Heredia had a rate of 91, within which canton rates were: San Pablo (131), Santo Domingo (130), downtown Heredia (100), Barva (97), and San Rafael (95). Alajuela, ranked fourth at a rate of 82, included the cantons Atenas (125), Alfaro Ruiz (123), Orotina (120), Naranjo (101), and Grecia (98). Guanacaste ranked fifth at a rate of 84. It includes Nandayure (181), Tilaran (106), Bagaces (93), Liberia (91), and Nicoya (84). Sixth place, Puntarenas had a rate of 68, and included the cantons Montes de Oro (164), Esparza (91), downtown Puntarenas (78), Golfito (73), and Aguirre (70). Finally, Limón had a rate of 57, including Siquirres (67), Pococi (63), Downtown Limón (59), Guacimo (58), and Talamanca (44).

## 4. Discussion

The current study provides a comprehensive overview of the cancer incidence and mortality trends in Costa Rica, shedding light on the burden of cancer within the country and across Central America. Our findings reveal an increase in cancer incidence rates in Costa Rica since the 1990s, with the country having the highest incidence in Central America in 2020. While the increase in incidence rates can be attributed to several factors, such as better diagnostic procedures, changes in risk factors, and an aging population, it is crucial to identify and address the underlying factors driving these trends.

In both men and women, the most common cancer types in Costa Rica were prostate and breast cancer, respectively, with non-melanoma skin cancer and colorectal cancer also showing increasing trends. These findings align with global patterns, where breast and prostate cancers are among the most common cancer types worldwide (Bray et al., 2018). The increasing trend of non-melanoma skin cancer may be partly attributed to increased sun exposure, changes in lifestyle, and improved diagnosis (Lallas et al., 2013).

Notably, our analysis indicates a decreasing trend in stomach and cervical cancer incidence, which may be the result of improved screening programs and public health interventions, such as the Human Papillomavirus (HPV) vaccination program (Herrero et al., 2015). The decline in lung cancer incidence, particularly among men, could be associated with successful tobacco control policies implemented in Costa Rica (Guerrero-López et al., 2013).

The analysis of cancer incidence by age group highlights the importance of targeted cancer prevention and control efforts across different age groups. For instance, the high incidence of hematopoietic and lymphatic cancers among individuals aged < 25 years calls for the development of specific strategies to manage these cancers in younger populations, including early detection and targeted therapies (Ward et al., 2014). Similarly, the increasing incidence of prostate cancer among men aged > 60 years underscores the need for age-specific screening and prevention strategies (Pinsky & Kramer, 2017).

Our findings on cancer mortality reveal that, while there have been declines in the mortality rates for certain cancer types, such as stomach, cervical, and lung cancers, other cancer types have seen increases in mortality, including colorectal and liver cancers. The reduction in stomach and cervical cancer mortality may be related to the implementation of effective screening programs and public health interventions, including the HPV vaccination program (Herrero et al., 2015). The decline in lung cancer mortality could be a result of successful tobacco control policies (Guerrero-López et al., 2013).

However, the increase in colorectal cancer mortality highlights the need for improved prevention and early detection strategies for this cancer type, as well as effective treatment approaches. The rise in liver cancer mortality may be associated with an increase in risk factors, such as viral hepatitis infections, alcohol consumption, and obesity (Makarova-Rusher et al., 2016).

Our study also revealed geographical disparities in cancer incidence and mortality across the provinces of Costa Rica. These disparities may be driven by various factors, including differences in risk factor exposure, access to healthcare, and socioeconomic status. Identifying and addressing these disparities is essential for developing targeted cancer prevention and control strategies and ensuring equitable access to healthcare services.

## 5. Conclusion

In summary, this study highlights the significant variations in cancer incidence and mortality trends across regions and countries, reflecting diverse risk factors, population demographics, and healthcare systems. The analysis incorporates data from multiple studies (Lallas et al., 2013; Bray et al., 2018; Herrero et al., 2015; Makarova-Rusher et al., 2016; Pinsky & Kramer, 2017; Guerrero-López et al., 2013; Ward et al., 2014), providing valuable insights into the global landscape of cancer epidemiology.

It is important to note that this study combines data from Costa Rica with available projections from the World Health Organization (WHO), enabling a comprehensive assessment of the current status of cancer. This integration of Costa Rican data and WHO projections allows for a more robust understanding of cancer incidence and mortality patterns.

A substantial proportion of the global cancer burden can be attributed to modifiable lifestyle and environmental factors, such as tobacco use, alcohol consumption, poor diet, physical inactivity, and exposure to carcinogens. Implementing effective prevention strategies targeting these risk factors is crucial to reduce cancer incidence and improve public health outcomes. Moreover, advancements in early detection, diagnosis, and treatment have contributed to improved survival rates for various cancer types. However, persistent disparities in healthcare access and the adoption of optimal cancer management practices in certain regions contribute to the observed variations in cancer outcomes.

Continued efforts to collect and analyze cancer epidemiological data are essential for informing public health policy, resource allocation, and research priorities. Collaborative initiatives among researchers, clinicians, policymakers, and public health organizations are crucial to address the global cancer burden and achieve equitable cancer care worldwide. By fostering a comprehensive understanding of the factors influencing cancer incidence and mortality, interventions can be tailored, and resources can be optimized to ultimately reduce the impact of this devastating disease on individuals, families, and societies globally.

## Strengths and Limitations

This study provides important insights into the burden of cancer in Costa Rica, including the incidence and mortality rates, as well as the variations in cancer incidence and mortality rates by geographic region and age group. The study has several strengths, including the use of national cancer registry data, which ensures the accuracy and reliability of the results. Additionally, the study provides a comprehensive analysis of the trends in cancer incidence and mortality rates, which can inform the development of targeted prevention and screening programs.

However, the study also has some limitations. First, the study only includes data up to 2020, which may not reflect the current situation of cancer in Costa Rica. Second, the study does not provide information on the socioeconomic and lifestyle factors that may contribute to the burden of cancer in the population. Finally, the study does not provide information on the access to healthcare services, which is an important factor in cancer prevention and treatment.

## References

[R1] Bray F, Ferlay J, Laversanne M, Brewster DH, Gombe Mbalawa C, Kohler B, Piñeros M, Steliarova-Foucher E, Swaminathan R, Antoni S, Soerjomataram I, & Forman D (2015). Cancer Incidence in Five Continents: Inclusion Criteria, Highlights from Volume X and the Global Status of Cancer Registration. International Journal of Cancer, 137, 2060–2071. 10.1002/ijc.29670 26135522

[R2] Bray F, Ferlay J, Soerjomataram I, Siegel RL, Torre LA, & Jemal A (2018). Global Cancer Statistics 2018: GLOBOCAN Estimates of Incidence and Mortality Worldwide for 36 Cancers in 185 Countries. CA: A Cancer Journal for Clinicians, 68, 394–424. 10.3322/caac.21492 30207593

[R3] Guerrero-López CM, Muños-Hernández JA, Sáenz de Miera-Juárez B, & Reynales-Shigematsu LM (2013). Consumo de tabaco, mortalidad y política fiscal en México [Tobacco Consumption, Mortality and Fiscal Policy in Mexico]. Salud Pública de México, 55, S276–S281. (In Spanish) 10.21149/spm.v55s2.5125 24626704

[R4] Herrero R, González P, & Markowitz LE (2015). Present Status of Human Papillomavirus Vaccine Development and Implementation. The Lancet Oncology, 16, e206–e216. 10.1016/S1470-2045(14)70481-4 25943065

[R5] International Agency for Research on Cancer (IARC) (2020). Cancer Today. https://gco.iarc.fr/today/home

[R6] International Agency for Research on Cancer (IARC) (n.a.). CI5—Cancer Incidence in Five Continents. https://ci5.iarc.fr/Default.aspx

[R7] Kwon H, & Kim K (2021). Diagnostic Accuracy of Transrectal Ultrasound-Guided Prostate Biopsy for Prostate Cancer: A Systematic Review and Meta-Analysis. Annals of Family Medicine, 19, 43–49.

[R8] Lallas A, Zalaudek I, Argenziano G, Longo C, Moscarella E, Di Lernia V, Al Jalbout S, & Apalla Z (2013). Dermoscopy in General Dermatology. Dermatologic Clinics, 31, 679–694. 10.1016/j.det.2013.06.008 24075553

[R9] Lindsay BR, Felger JC, Fischer CS, Meza R, & Ríos-Zertuche D (2020). Barriers to Cancer Screening Participation in Costa Rica: A Qualitative Study. PLOS ONE, 15, e0239266.33035213

[R10] Makarova-Rusher OV, Altekruse SF, McNeel TS, Ulahannan S, Duffy AG, Graubard BI, Greten TF, & McGlynn KA (2016). Population Attributable Fractions of Risk Factors for Hepatocellular Carcinoma in the United States. Cancer, 122, 1757–1765. 10.1002/cncr.29971 26998818 PMC5548177

[R11] Ministerio de Salud (2021). Instituto Nacional de Estadística y Censos. https://www.ministeriodesalud.go.cr/index.php/estadisticas-nacionales/instituto-nacional-de-estadistica-y-censos-inec

[R12] Pinsky PF, Prorok PC, & Kramer BS (2017). Prostate Cancer Screening—A Perspective on the Current State of the Evidence. The New England Journal of Medicine, 376, 1285–1289. 10.1056/NEJMsb1616281 28355509

[R13] Waks AG, Winer EP, & Kurian AW (2021). Genetic Testing and Counseling for Hereditary Breast Cancer in the Era of Panel Testing and Multigene Panels. JAMA Oncology, 7, 204–211.

[R14] Ward E, DeSantis C, Robbins A, Kohler B, & Jemal A (2014). Childhood and Adolescent Cancer Statistics, 2014. CA: A Cancer Journal for Clinicians, 64, 83–103. 10.3322/caac.21219 24488779

[R15] WHO World Health Organization (2010). International Classification of Diseases for Oncology (3rd ed.). World Health Organization. https://apps.who.int/iris/handle/10665/96612

[R16] WHO World Health Organization (2021). Cancer. World Health Organization. https://www.who.int/health-topics/cancer#tab=tab_1

[R17] Williams GR, Dunlop MG, Carroll RJ, & Campbell PT (2021). Association of Colorectal Cancer Risk with Polymorphisms in Cancer Susceptibility Genes. JAMA Oncology, 7, 190–197.

[R18] Zhang C, Chen Y, Zhou B, Li Y, Chen X, Zhang X, & Li Y (2021). The Relationship between Circulating Tumor Cells and Survival in Patients with Advanced Colorectal Cancer: A Systematic Review and Meta-Analysis. Frontiers in Oncology, 11, Article ID: 793311. 10.3389/fonc.2021.671874

